# Breast Reconstruction Using the Axillary-Approach Endoscopic Extended Latissimus Dorsi (Ax-eeLD) Flap

**DOI:** 10.3390/jcm15020703

**Published:** 2026-01-15

**Authors:** Shinsuke Akita, Yoshihisa Yamaji, Haruka Maei, Kahoko Yamada, Nobuhiro Ando, Kentaro Kosaka, Hiroshi Fujimoto, Nobuyuki Mitsukawa

**Affiliations:** 1 Department of Plastic and Reconstructive Surgery, Graduate School of Medicine, Chiba University, Chiba 260-8670, Japan; 2 Department of General Surgery, Graduate School of Medicine, Chiba University, Chiba 260-8670, Japan

**Keywords:** breast reconstruction, endoscopic latissimus dorsi flap, breast hypoplasia, breast cancer, BREAST-Q

## Abstract

**Background/Objectives**: Although the endoscopic extended latissimus dorsi (eeLD) flap avoids dorsal scarring, a lateral thoracic incision is still required. We developed an axillary-approach endoscopic extended latissimus dorsi (Ax-eeLD) flap enabling harvest through a single 40-mm axillary incision and two 5-mm ports. This study evaluated its safety and feasibility and compared outcomes with conventional eeLD. **Methods**: Patients who underwent Ax-eeLD flap (study group) were retrospectively analyzed and compared with the patients who underwent conventional eeLD flap (control group, *n* = 15). The flap was elevated endoscopically via a single 40-mm axillary incision and two 5-mm ports, harvesting the entire latissimus dorsi muscle with its surrounding adipose tissue. Outcomes included incision length, operative time, complications, secondary fat grafting, and BREAST-Q scores. **Results**: Fifteen patients (post-mastectomy, *n* = 13; congenital hypoplasia, *n* = 2) underwent Ax-eeLD flap. All procedures used only the planned incisions without intraoperative complications. The study group had significantly shorter incisions than the control group (39 ± 1 mm vs. 89 ± 9 mm, *p* < 0.01). Operative times were similar between the groups. Eight patients developed seromas, all of which were resolved by outpatient aspiration. The frequency of postoperative cases requiring fat grafting did not differ significantly between the study and control groups (4 vs. 8; *p* = 0.26). BREAST-Q scores improved postoperatively and were similar between groups. **Conclusions**: Ax-eeLD flap enables minimally invasive harvest of the latissimus dorsi without lateral thoracic scarring. This retrospective case series supports technical feasibility and safety; further prospective studies with objective volume assessment are required.

## 1. Introduction

The latissimus dorsi (LD) flap has long been used as a reliable option for breast reconstruction owing to its consistent vascularity and procedural safety [1]. However, the conventional technique requires a long dorsal incision, often resulting in a conspicuous and aesthetically unfavorable scar [2,3,4]. To overcome this limitation, we previously developed an endoscope-assisted extended LD (eeLD) flap combined with lipofilling, enabling reconstruction without a dorsal scar (conventional eeLD flap) [5]. However, this approach still required a lateral thoracic incision for flap elevation, leaving a visible chest wall scar.

With the recent spread of endoscopic and robotic nipple-sparing mastectomy (NSM), mastectomy can be performed through a small axillary incision alone [6,7]. As axillary-access surgery becomes more common, the need for reconstructive techniques compatible with the same incision has increased correspondingly [8]. Furthermore, in congenital breast hypoplasia, such as Poland syndrome, no mastectomy incision exists, making inconspicuous scarring highly desirable [9]. Axillary incisions are associated with high patient satisfaction among young Asian women undergoing aesthetic breast surgery [10,11]. Thus, developing an extended LD flap harvested entirely through a single axillary incision is increasingly relevant.

**Figure 1:**
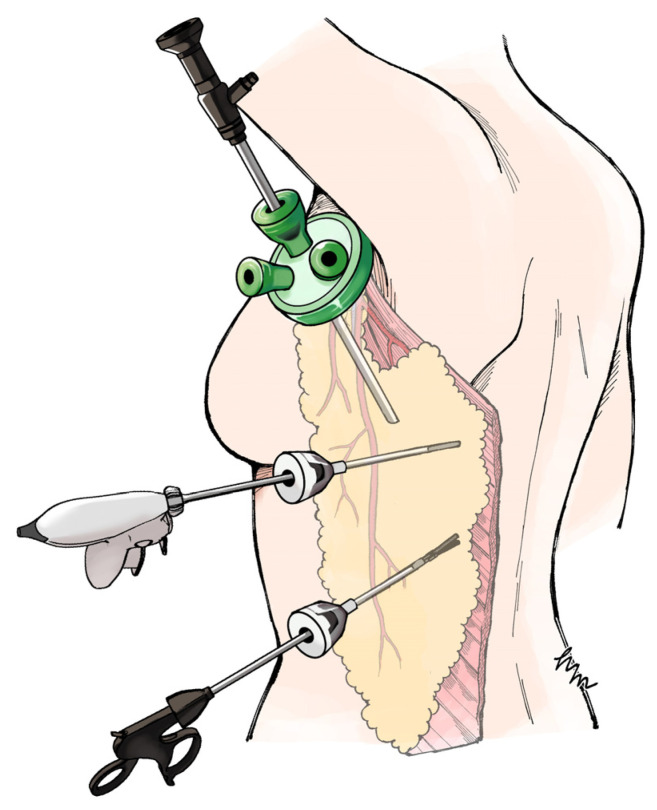
Schematic overview of the axillary-approach endoscopic extended latissimus dorsi (Ax-eeLD) flap. Multiple devices can be inserted through the 40-mm port, while the caudal 5-mm port allows insertion of a single device. To visualize the entire latissimus dorsi muscle and elevate the muscle–fat flap, the use of two 5-mm ports facilitates the procedure.

We developed the axillary-approach extended endoscopic latissimus dorsi (Ax-eeLD) flap, which enables flap elevation through a 40-mm axillary incision with two 5-mm ports, without additional anterior or lateral thoracic scarring (Figure 1).

The primary objective of this study was to verify the safety and feasibility of this procedure. The secondary objective was to determine whether the Ax-eeLD flap could maintain patient satisfaction comparable to that of the conventional eeLD flap despite the shorter incision.

## 2. Materials and Methods

### 2.1. Ethical Approval

This study was approved by the Institutional Review Board of the Graduate School of Medicine, Chiba University (approval no. M10473). Written informed consent was obtained from all participants.

### 2.2. Study Design and Patient Selection

This retrospective case–control study included patients who underwent breast reconstruction using the Ax-eeLD flap between January 2023 and April 2025. Data was collected for all patients treated with this technique. For comparison, previously published data on patients who underwent conventional eeLD flap reconstruction were used; all patients provided additional consent for data reuse.

All Ax-eeLD procedures were performed by the same surgical team that had established and routinely performed the conventional eeLD flap, ensuring technical consistency. Indications included breast reconstruction following mastectomy for breast cancer or correction of congenital breast hypoplasia. Exclusion criteria for post-mastectomy patients included the presence of a lateral thoracic incision suitable for conventional eeLD flap elevation and a history of radiation therapy that could compromise skin elasticity. No exclusion criteria were applied to patients with congenital breast hypoplasia.

### 2.3. Preoperative Preparation

In patients with an insufficient skin envelope, either following mastectomy or due to congenital hypoplasia, preoperative tissue expansion was performed. When sufficient skin redundancy remained after mastectomy, reconstruction was performed without tissue expansion by advancing the caudal skin flap cranially to recreate the inframammary fold, thereby enabling single-stage reconstruction.

### 2.4. Surgical Technique

**Figure 2:**
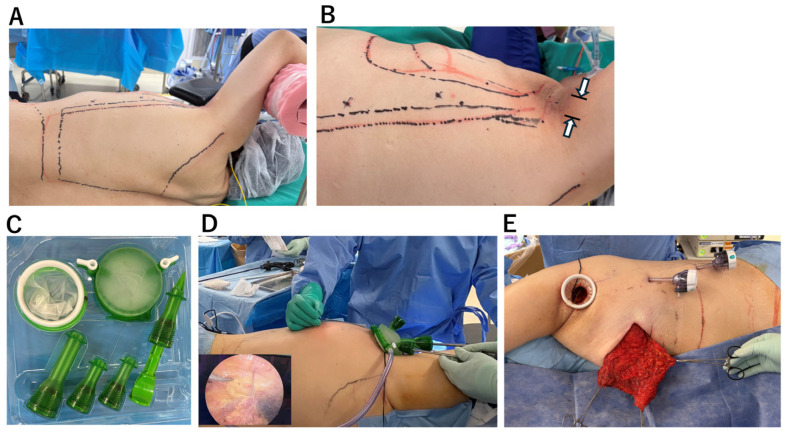
Intraoperative procedural steps. (**A**) Location of the 40-mm axillary skin incision and planned 5-mm port insertion sites. (**B**) The upper limb is not fully immobilized, allowing repositioning according to the camera angle. (**C**) Single-port endoscopic surgery platform: GelPOINT Mini Advanced Access Platform (Applied Medical, Rancho Santa Margarita, CA, USA). (**D**) When the target area is not clearly visible within the camera field of view, percutaneous insertion of a 23-gauge needle can be used for orientation. (**E**) The muscle–fat flap is mobilized through the subcutaneous tissue to the anterior chest. In cases with preexisting skin incisions or scars on the chest, these sites are opened to allow extraction of the flap.

All procedures were performed with the patient placed in the lateral decubitus position (Figure 2A). A 40-mm axillary skin incision was made (Figure 2B).

Under direct vision, the insertion of the LD muscle was divided, and the thoracodorsal vessels and thoracodorsal nerve were looped with vessel tapes to facilitate identification during endoscopic dissection (Supplemental Digital Content, Video S1). After creating sufficient subcutaneous and submuscular working space for endoscopic manipulation, the GelPOINT Mini^®^ access platform (Applied Medical, Rancho Santa Margarita, CA, USA) was introduced to establish a single-site access system (Figure 2C). When the GelPOINT Mini^®^ was not available, a wound retractor (Alexis^®^ Wound Retractor, Applied Medical, Rancho Santa Margarita, CA, USA) in combination with a sterile surgical glove was used as an alternative access platform. Carbon dioxide was insufflated through one port to expand the subcutaneous space, maintaining stable pressure at 10 mmHg to ensure adequate room for endoscopic instruments. The optimal viewing point for endoscopic observation was confirmed by percutaneously advancing a 23-gauge needle. Based on this, an additional 5-mm port was inserted at a site that provided effective visualization and facilitated precise dissection (Figure 2D). The extended LD flap—comprising the entire latissimus muscle and the superficial fat layer adherent to the deep fascia of the lateral thorax—was harvested in continuity. Because the flap could not be exteriorized through the 40 mm axillary incision, it was transferred subcutaneously into a pre-created pocket on the anterior chest wall to form the breast mound (Figure 2E). The coagulation dissection system and endoscopic ports were disposable. While endoscopic port prices range from inexpensive to relatively high, the total cost of disposable instruments per case was approximately ¥110,000 (approximately US $700).

Fat grafting was performed as needed, using less than 150 mL of fat harvested by liposuction from the abdomen or thighs. The aspiration was centrifuged at 1200 *g* for 3 min, after which the fluid and blood components were removed. In cases requiring fixation of the breast mound and lacking a chest wall incision, fixation sutures were placed endoscopically using 3-0 Vicryl^®^ (Ethicon, LLC, Guaynabo, PR, USA). Two negative-pressure drains were placed through the 5-mm port sites—one in the back and one in the chest—utilizing the port wounds as drain exit sites. Drains were removed when output was <20 mL/day or when 2 weeks had passed.

### 2.5. Evaluation of Outcomes

The primary outcome was to determine whether the Ax-eeLD flap proposed in this study could be safely completed without intraoperative skin incision extension or surgical procedure modification, and whether postoperative complications occurred. Furthermore, we compared treatment outcomes of the Ax-eeLD flap (study group) with those of the conventional eeLD flap reported in a previous study as the historical control group [5]. We compared the two groups with respect to skin incision length, operative time, and BREAST-Q scores for satisfaction with breasts, satisfaction with back, and back well-being at 3 months or later postoperatively [12,13]. These endpoints were selected because our previous study had demonstrated that the conventional eeLD flap yielded significantly superior aesthetic and patient-reported outcomes compared with the traditional LD flap with dorsal skin incision. This study demonstrated that the Ax-eeLD flap can achieve less conspicuous scarring without disadvantage, at least compared with the conventional eeLD flap. As a reference, in delayed reconstruction cases and breast hypoplasia patients, the improvement from the preoperative score was also recorded.

### 2.6. Statistical Analysis

All statistical analyses were performed using JMP version 13 (SAS Institute, Cary, NC, USA). Categorical variables were compared using the chi-square test or Fisher’s exact test, as appropriate. Continuous variables were compared using Student’s *t*-test after confirmation of normality. A *p*-value < 0.05 was considered statistically significant.

## 3. Results

A total of 15 breasts in 15 patients underwent breast reconstruction using the Ax-eeLD flap. Among them, 13 patients received post-mastectomy breast reconstruction, and 2 patients had congenital breast hypoplasia. The mean age and BMI were 41.1 ± 12.4 years and 21.1 ± 2.2 kg/m^2^, respectively. All patients were non-smokers. In 10 post-mastectomy cases requiring an additional skin envelope, as well as in both of the 2 hypoplastic breast cases, preoperative tissue expansion was performed, whereas the remaining 3 post-mastectomy patients did not require expansion due to adequate residual skin on the chest wall. The flap weight could not be measured in this case series because the skin incision was minimal and the skin paddle was not exteriorized during surgery. The mean volume of fat injection was 122 ± 26 mL. All procedures were completed through the planned axillary incision without additional scarring on the back or lateral thorax, and no remarkable intraoperative complications were observed. There was no significant difference in operative time between the study group (*n* = 15) and the control group (*n* = 13) (322.1 ± 31.1 min vs. 331.5 ± 38.1 min; *p* = 0.40), (Table 1).

In the postoperative course, seroma occurred in 8 patients, all of whom were successfully managed with outpatient aspiration (1.9 ± 1.2 sessions, range 0–3). There was no difference in BMI between the group with seroma (*n* = 8) and the group without seroma (*n* = 7) (21.5 ± 1.5 vs. 20.6 ± 1.8, *p* = 0.46 in *t*-test). No other significant postoperative complications were observed. Reduction in breast mound volume stabilized by approximately 8 weeks postoperatively. The proportion of patients requiring secondary fat grafting did not differ between the study and control groups (4 of 15 vs. 8 of 15; *p* = 0.26, Fisher’s exact test) (Table 1). The length of the skin incision scar in study group cases (*n* = 15) was significantly shorter than in the 9 cases of the control group where an incision was placed on the lateral chest (39 ± 1 mm vs. 89 ± 9 mm; *p* < 0.01). Overall, postoperative outcomes were comparable to those of the conventional eeLD flap, indicating that the smaller incision area and length did not adversely affect flap viability or fat absorption.

Postoperative BREAST-Q scores in all cases that underwent Ax-eeLD as delayed breast reconstruction for breast cancer or breast reconstruction for breast hypoplasia improved significantly from 30.4 ± 12.1 to 78.3 ± 8.9 (*n* = 9, *p* = 0.01). Postoperative scores for satisfaction with breast, satisfaction with back, and back well-being showed no difference between the study group and control group. In summary, the Ax-eeLD flap could be safely performed through a smaller skin incision without compromising operative time, postoperative course, or satisfaction with the breast or donor site compared to the conventional eeLD flap.

**Figure 3:**
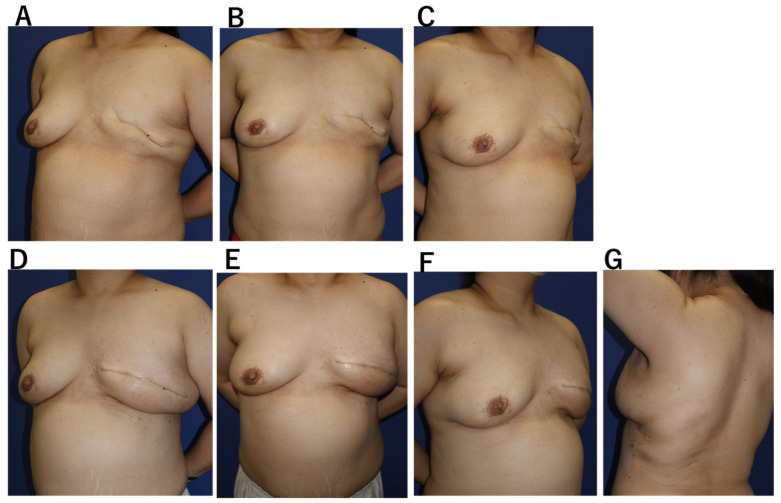
Case 1. A 53-year-old woman after left total mastectomy. Because sufficient skin redundancy was present, single-stage autologous breast reconstruction was performed using circumferential skin lifting and an Ax-eeLD flap. (**A**–**C**) Preoperative breast photographs: (**A**) right anterior oblique view, (**B**) frontal view, and (**C**) left oblique view. (**D**–**G**) Postoperative photographs: (**D**) right anterior oblique view, (**E**) frontal view, (**F**) left oblique view, and (**G**) donor site.

**Case 1.** A 53-year-old woman with bilateral breast cancer had previously undergone breast-conserving surgery on the right breast and mastectomy on the left breast two years earlier (Figure 3). Because the prior incision was limited to the peri areolar area and the remaining skin envelope showed adequate laxity, single-stage reconstruction was planned without tissue expansion, using caudal flap advancement to redefine the inframammary fold (4). A 4-cm axillary incision was used to harvest the LD flap, which was transferred subcutaneously to the anterior chest and supplemented with 200 mL of fat grafting. The drain was removed on the 14th postoperative day. A seroma developed on the donor site and resolved after three aspirations. Breast volume stabilized by 8 weeks, with satisfactory symmetry and no donor-site functional impairment.

**Figure 4:**
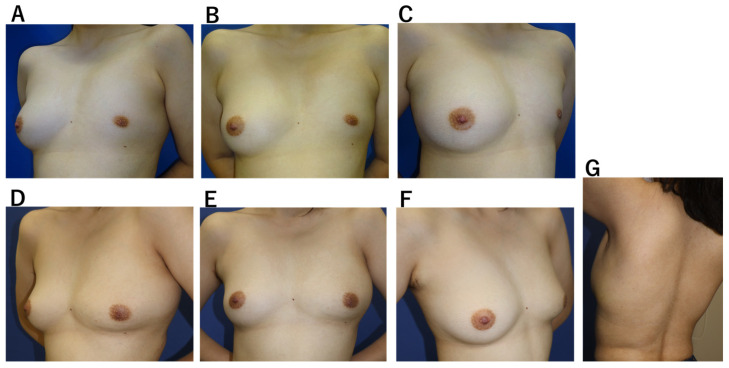
Case 2. A 20-year-old woman with left breast hypoplasia. Because of insufficient skin and marked asymmetry of the nipple–areola complex, skin expansion was first performed using a tissue expander inserted through an axillary incision, followed by reconstruction with an Ax-eeLD flap. (**A**–**C**) Preoperative breast photographs: (**A**) right anterior oblique view, (**B**) frontal view, and (**C**) left oblique view. (**D**–**G**) Postoperative photographs: (**D**) right anterior oblique view, (**E**) frontal view, (**F**) left oblique view, and (**G**) donor site.

**Case 2.** A 20-year-old woman with idiopathic breast hypoplasia, without pectoralis major deficiency (Figure 4). Mild scoliosis was present, but no pectus excavatum. A tissue expander was inserted through a 40-mm axillary incision and preoperatively expanded. Using the same incision, an LD flap was subsequently harvested and combined with 150 mL of fat grafting. The drain was removed on the 14th postoperative day, and only one aspiration was required for a minor seroma, which resolved without complication. Breast volume stabilized by 8 weeks, and no functional donor-site impairment was observed.

## 4. Discussion

Endoscopic LD flap harvesting has long been described as a minimally invasive alternative to conventional LD reconstruction; however, its clinical use has generally been confined to partial breast reconstruction or implant-assisted procedures because of limited available autologous volume [4,14,15,16,17]. In more recent reports, the adjunctive use of fat grafting with LD flaps has been proposed as a means to compensate for this limitation, allowing selected cases of total breast reconstruction without implants and thereby suggesting a gradual expansion of the reconstructive indications of endoscopic LD–based techniques [5,18,19,20]. This study presents a novel technique for harvesting the extended endoscopic latissimus dorsi flap (Ax-eeLD flap) via a 40-mm axillary incision. The key feature of this approach is that it enables safe and efficient elevation of the flap without creating additional scars on the back or lateral thorax. The concept of the eeLD flap is based on elevating the entire latissimus dorsi muscle—fully detached from its origin—together with the anterior superficial fat layer supplied by the thoracodorsal vessels and combining it with lipofilling to achieve adequate volume for post-mastectomy reconstruction [5]. In anticipation of the increasing adoption of robot-assisted mastectomy, this report confirms that flap elevation is feasible through an incision as small as 40 mm, consistent with recent advances in minimally invasive breast surgery [6,7].

Compared to the lateral thoracic incision used in conventional Ax-eeLD flaps, the axillary incision offers the advantage of enabling safe and reliable axillary manipulation during surgery. The vascular pedicle can be directly visualized and securely preserved prior to endoscopic dissection. Furthermore, dissection of the latissimus dorsi muscle attachment under direct visualization enabled the complete transfer of the elevated muscle-fat flap to the anterior chest wall. These factors allowed the Ax-eeLD flap to be completed safely without prolonging the operative time, despite its skin incision being significantly shorter than that of the conventional eeLD flap. The thoracodorsal artery, a major vascular pedicle, was secured under direct visualization, and the perforating branches from the intercostal arteries were reliably visible within the endoscopic field of view. Under CO_2_ insufflation, our case series did not experience massive bleeding that obstructed the field of view or made control difficult.

We used the patient-reported outcome measure BREAST-Q to compare treatment outcomes, providing a standardized approach for assessing both aesthetic and functional satisfaction across diverse patient groups [12,13]. Breast reconstruction outcomes are influenced by multiple factors, including the surgical technique, scar location, disease-specific deformities, and whether nipple–areolar complex reconstruction is performed. As the primary aim of this study was to verify the safety of our newly developed technique, the procedure was applied to patients with different clinical backgrounds, including immediate and delayed breast reconstruction following mastectomy, and congenital breast hypoplasia. Aesthetic challenges and treatment goals vary depending on individual conditions and patient preferences. In Case 1, the patient did not desire nipple–areolar reconstruction and had sufficient residual skin on the affected side. Therefore, delayed reconstruction was performed without a tissue expander, using an abdominal advancement flap and Ax-eeLD, resulting in a satisfactory outcome [21]. In Case 2, involving congenital hypoplasia, asymmetry in areolar diameter strongly influences subjective cosmetic perception [9]. To address this, we discussed the patient’s preferences and incorporated preoperative tissue expansion to increase the areolar diameter prior to surgery. Given the variability in patient background and treatment goals in this study, subjective patient-reported outcomes were deemed appropriate for evaluating treatment effects. While non-inferiority to conventional eeLD was demonstrated, no clear advantage of Ax-eeLD in subjective evaluation was observed, despite its shorter scar length. This may be due to the limited number of BREAST-Q items specifically assessing scar-related concerns. For future research aimed at demonstrating the cosmetic superiority of Ax-eeLD, incorporating scar-focused tools, such as the SCAR-Q, could be beneficial [22].

In our case series, no hematomas were observed; however, seromas requiring outpatient drainage were observed in 8 of 15 cases. The use of quilting sutures and fibrin sealant has been shown to be beneficial in preventing seromas in expanded latissimus dorsi flaps [23]. Future studies should test the usefulness of these techniques.

Although eeLD flaps can compensate for postoperative volume loss through fat grafting or adjunctive fat grafting, they inherently provide less volume compared to conventional LD flaps harvested via dorsal skin incisions [5]. This study aimed to minimize postoperative tissue loss by expanding skin coverage with tissue expanders or by advancing caudal anterior chest skin flaps cephalad, thereby reducing compressive forces on the grafted tissue. However, secondary fat grafting was required in 4 out of 15 cases even with the Ax-eeLD flap. Therefore, the temporal shrinkage of the grafted tissue remains an issue requiring further investigation. An interesting new finding in this study is that, in some patients, including the patient presented with Case 1, satisfactory symmetry was achieved in a single operation without the use of tissue expanders. This suggests that adequate soft tissue envelope capacity may help mitigate postoperative volume reduction. Changes in flap volume and the potential for additional fat grafting are critical considerations in this procedure, necessitating careful evaluation. However, the relatively small axillary incision used in this approach makes it difficult to temporarily expose the harvested flap outside the body, precluding accurate intraoperative assessment of graft volume. This study is a feasibility study of the Ax-eeLD flap, which was found to be safe and without major adverse events. In this study, it was not possible to compare flap volume between the Ax-eeLD flap and the conventional eeLD flap. Future validation studies would benefit from incorporating objective, quantitative volume measurement techniques, such as those using imaging modalities.

This study has several limitations. First, Ax-eeLD was performed mainly in patients with relatively small breast volume relative to body size. Patients requiring larger volumes may exceed flap capacity, and clear selection criteria have not yet been established. Further case accumulation is necessary. Second, no cases involved immediate reconstruction after robotic/endoscopic NSM via axillary skin incision. New challenges may arise under such conditions, but robotic assistance may improve operative maneuverability. Future studies should evaluate these settings to further advance minimally invasive breast reconstruction.

## 5. Conclusions

The Ax-eeLD flap enables safe elevation of an extended LD flap through a 40 mm axillary incision, avoiding visible dorsal or lateral scarring without prolonging operative time or compromising patient-reported outcomes. This retrospective case series supports technical feasibility and safety; further prospective studies with objective volume assessment are required.

## Figures and Tables

**Table 1:** Comparison of an axillary-approach endoscopic extended latissimus dorsi flap (study group) and a conventional endoscopic extended latissimus dorsi flap (control group).

	Study Group (*n* = 15)	Control Group (*n* = 15)	*p* Value
Age (years old)	41.1 ± 12.4	46.7 ± 8.4	0.15
BMI (kg/m^2^)	21.1 ± 2.2	20.8 ± 1.4	0.72
Operation Time (min)	322.1 ± 31.1	331.5 ± 38.1 (unilateral cases, *n* = 13)	0.48
Number of Punctures after drain removal	1.9 ± 1.2	2.1 ± 1.0	0.63
Number of breasts treated with secondary fat injection	4	8	0.26
Length of postoperative scar (mm)	39 ± 1	89 ± 9 (on the lateral chest, *n* = 9)	<0.001
BREAST-Q Scores			
Satisfaction with Breast	78.3 ± 9.2	77.3 ± 11.1	0.80
Satisfaction with Back	83.5 ± 8.2	82.8 ± 9.6	0.84
Back Well-being	87.3 ± 8.1	85.3 ± 8.9	0.54

## Data Availability

The data presented in this study are available on reasonable request from the corresponding author. The data are not publicly available due to privacy and ethical restrictions involving patient information.

## Supplementary Materials

The following supporting information can be downloaded at: https://www.mdpi.com/article/10.3390/jcm15020703/s1. Video S1: Axillary Approach Endoscopic Extended LD Flap Elevation Procedure.

## Abbreviations

The following abbreviations are used in this manuscript:

eeLD	endoscopic extended latissimus dorsi
LD	latissimus dorsi
Ax-eeLD	axillary-approach endoscopic extended latissimus dorsi
BMI	body mass index
NSM	Nipple-Sparing Mastectomy
